# Cholesterol in the Viral Membrane is a Molecular Switch Governing HIV‐1 Env Clustering

**DOI:** 10.1002/advs.202003468

**Published:** 2020-12-21

**Authors:** Jon Ander Nieto‐Garai, Aroa Arboleya, Sara Otaegi, Jakub Chojnacki, Josefina Casas, Gemma Fabriàs, F‐Xabier Contreras, Hans‐Georg Kräusslich, Maier Lorizate

**Affiliations:** ^1^ Instituto Biofisika (UPV/EHU, CSIC) University of the Basque Country Leioa E‐48940 Spain; ^2^ Fundación Biofísica Bizkaia/Biofisika Bizkaia Fundazioa (FBB) Barrio Sarriena s/n Leioa E‐48940 Spain; ^3^ Department of Biochemistry and Molecular Biology Faculty of Science and Technology University of the Basque Country Leioa E‐48940 Spain; ^4^ IrsiCaixa AIDS Research Institute Badalona 08916 Spain; ^5^ Research Unit on BioActive Molecules. Department of Biological Chemistry Institute for Advanced Chemistry of Catalonia (IQAC‐CSIC) Barcelona Catalonia 08034 Spain; ^6^ Liver and Digestive Diseases Networking Biomedical Research Center (CIBEREHD) ISCII Madrid 28029 Spain; ^7^ Ikerbasque Basque Foundation for Science Bilbao 48013 Spain; ^8^ Department of Infectious Diseases Virology Universitätsklinikum Heidelberg Heidelberg 69120 Germany

**Keywords:** cholesterol, Env clustering, gp41 cytoplasmic tails, HIV‐1, protein–lipid interactions

## Abstract

HIV‐1 entry requires the redistribution of envelope glycoproteins (Env) into a cluster and the presence of cholesterol (chol) in the viral membrane. However, the molecular mechanisms underlying the specific role of chol in infectivity and the driving force behind Env clustering remain unknown. Here, gp41 is demonstrated to directly interact with chol in the viral membrane via residues 751–854 in the cytoplasmic tail (CT_751–854_). Super‐resolution stimulated emission depletion (STED) nanoscopy analysis of Env distribution further demonstrates that both truncation of gp41 CT_751–854_ and depletion of chol leads to dispersion of Env clusters in the viral membrane and inhibition of virus entry. This work reveals a direct interaction of gp41 CT with chol and indicates that this interaction is an important orchestrator of Env clustering.

## Graphical abstract

The HIV‐1 fusion protein (Env) interacts with viral membrane cholesterol by the gp41 subunit cytoplasmic tail. This gp41–cholesterol interaction is the driving force behind the redistribution of Env into a cluster, a process necessary for fusion with the host cell. This mechanistic model sheds new light toward the molecular action of antiretroviral drugs targeting cholesterol in HIV‐1.

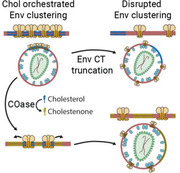

## 1 Introduction

HIV‐1 is surrounded by a lipid bilayer in which the HIV‐1 envelope glycoproteins (Env) are embedded. Env is composed of two subunits—gp120 or surface glycoprotein and gp41 or transmembrane glycoprotein—which associate into trimers. The transmembrane gp41 subunit is responsible for the virus‐to‐cell and cell‐to‐cell fusion in the HIV‐1 replication cycle, and it contains the epitopes for a wide variety of broadly neutralizing antibodies.^[^ ^1^, ^2^ ^]^ This makes gp41 a great target for the development of prophylactic vaccines against HIV‐1 and antiretroviral drugs targeting the entry of the virus into the host cell.

HIV‐1 particles contain between 7 and 14 Env trimers, a very low number compared to other enveloped viruses.^[^ ^3^, ^4^ ^]^ This property, which is thought to provide an evolutionary advantage to avoid detection by the immune system of the host organism^[^ ^5^, ^6^ ^]^ has in contrast a limiting effect on the fusion capacity of the virus. Membrane fusion is an energetically demanding process and in most viral strains a single Env trimer is not sufficient for inducing efficient viral entry.^[^ ^7^ ^]^ To ensure proper fusion, HIV‐1 seems to compensate for its low trimer number by inducing a redistribution of Env into a single focus upon virus maturation, in a process known as Env clustering. This clustering is thought to facilitate the coordinated action of several neighboring Env trimers and facilitate fusion.^[^ ^8^ ^]^ Although several factors have been shown to be important for clustering, the precise molecular mechanism governing this process has not yet been fully elucidated.

HIV‐1 acquires its lipid envelope from the plasma membrane of the producer cell,^[^ ^9^ ^]^ but the viral membrane shows significant differences both in composition and physicochemical properties compared to its donor membrane.^[^ ^10^, ^11^ ^]^ It is enriched in phosphatidylserine, sphingomyelin, hexosylceramide, saturated species of phosphatidylcholine,^[^ ^12^, ^13^ ^]^ and phosphatidylinositol (4,5) biphosphate.^[^ ^14^, ^15^ ^]^ Additionally, although not necessarily enriched, cholesterol (chol) constitutes up to 50% of the total lipid molecules in the viral membrane.^[^ ^12^, ^13^ ^]^ The high amount of saturated phosphoglycerolipids, sphingomyelin, and chol in the HIV‐1 membrane has led to its comparison with chol‐rich raft‐like nanodomains, an idea that is further supported by the ordered lateral membrane structure (l_o_ phase) found in HIV‐1.^[^ ^16^ ^]^ Several compounds that alter membrane properties such as its structure and rigidity have been shown to inhibit virus infectivity at the entry step,^[^ ^17^, ^18^, ^19^ ^]^ including lipidomimetic compounds that specifically alter membrane fluidity.^[^ ^20^ ^]^ These reports demonstrate that modulation of membrane fluidity and structures can be a valid approach for the development of antiretroviral compounds.^[^ ^21^ ^]^ Additionally, depletion^[^ ^22^, ^23^, ^24^, ^25^ ^]^ or sequestering^[^ ^26^, ^27^ ^]^ of chol—a known regulator of membrane structure—from the viral membrane has also been found to inhibit viral entry. However, the precise molecular mechanism underpinning the role of chol in viral infectivity has not been deciphered yet.

Chol and chol‐rich membrane nanodomains have been described to laterally self‐aggregate different proteins in the plasma membrane to regulate their function.^[^ ^28^, ^29^, ^30^ ^]^ Thus, the role of chol in infectivity could be related to Env distribution or function. Indeed, Env has been suggested to associate with raft‐like nanodomains applying indirect methods based on isolation of detergent resistant membranes (DRMs).^[^ ^31^, ^32^, ^33^, ^34^ ^]^ However, the study of protein–lipid interactions by DRMs relies on the use of low temperature and detergents,^[^ ^35^ ^]^ which can alter the natural structure of membranes and produce artifacts.^[^ ^36^ ^]^ Thus, they do not constitute direct proof of an interaction of gp41 with chol in the native viral membrane.

Here, we have found that gp41 directly interacts with chol in viral particles via residues 751–854 in its cytoplasmic tail (CT). Furthermore, we have shown by super‐resolution nanoscopy (STED) that both the gp41 CT_751–854_ and chol in the viral membrane are necessary for efficient clustering of Env and full entry competence. Altogether, these results support a mechanism by which interaction of gp41 with chol in the viral membrane drives lateral self‐aggregation and clustering of Env trimers, facilitating fusion with the host cell and consequent viral entry, revealing chol as an essential component of the molecular platform driving Env clustering.

## 2 Results

### 2.1 HIV‐1 gp41 Interacts with Chol in the Viral Membrane

**Figure 1:**
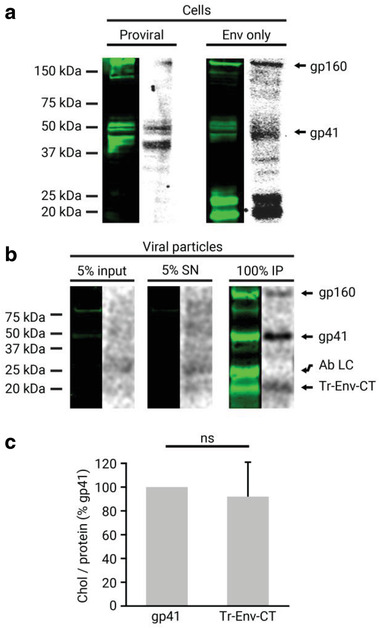
Gp41 interaction with chol in cells and released mature viral particles. a) Gp41 was immunoprecipitated from HEK 293T cells fed with the chol analogue [^3^H]‐photochol and detected by Western blot with the anti‐gp41 chessie‐8 antibody (green lanes). The interacting radioactively labeled lipid was detected by autoradiography of the same membrane (gray lanes). Cells were transfected either with a proviral pCHIV plasmid expressing all of the viral proteins or with an Env only expressing pCAGGS plasmid. b) Viral particles were purified from HEK 293T cells transfected with the pCHIV proviral plasmid and treated with the chol analogue [^3^H]‐photochol. Gp41 from input (5% of lysed viral particles), supernatant (SN; 5% of total), and immunoprecipitation (IP) from purified viral particles were detected by Western blot with the anti‐gp41 chessie‐8 antibody (green lane), and the interacting radioactively labeled lipid by autoradiography of the same membrane (gray lane). Protein bands corresponding to gp160, gp41, the light chain of the antibody used for immunoprecipitation (Ab LC), and a truncated form of gp41 (Tr‐Env‐CT) are labeled. c) Quantification of the chol/protein signal ratio of the Tr‐Env‐CT protein fragment compared to the full‐length gp41. The bars and whiskers represent the mean ± SD of experiments from five independent viral purifications (*n* = 5). Statistical significance was assessed by analysis of variance and Tukey test.

Chol constitutes up to 50% of the total lipids in the HIV‐1 viral membrane and is necessary for infectivity. Conceivably, chol may directly interact with viral membrane proteins and trigger HIV‐1 membrane protein activity. The main candidate for this is Env and specifically its transmembrane subunit gp41, the protein responsible for membrane fusion. To test if a direct interaction between Env and chol exists, we first studied the interaction of gp41 with membrane chol in virus‐producing cells using a well‐established photoaffinity binding strategy using a bifunctional photoactivatable and radioactively (tritiated) labeled chol analogue ([^3^H]‐photochol).^[^ ^36^ ^]^ A potential gp41–lipid interaction was studied when Env was expressed alone or in a proviral context to determine an influence of other viral proteins. HEK 293T were transfected with either a proviral pCHIV plasmid or an Env only expressing plasmid (Table S1, Supporting Information). Viral protein‐expressing cells were then incubated with the chol analogue [^3^H]‐photochol for 18 h to allow sufficient lipid capture and incorporation into the membranes (Figure S1, Supporting Information). Cells were irradiated with ultraviolet light, thereby cross‐linking proteins and lipids in close proximity (<3 Å). A comparison of the protein signal in the Western blot with the lipid signal in the autoradiography demonstrated that wild‐type gp41 directly interacted with chol in cells expressing all of the viral proteins (**Figure** **1**a, proviral). Notably, this interaction was also observed when Env was expressed alone (Figure 1a, Env only), revealing that the chol‐interacting properties are intrinsic to gp41 and do not require the presence of other viral proteins. When Env was expressed alone, two additional bands of ≈25 and ≈20 kDa recognized by the anti‐gp41 antibody were also found to interact with chol. As explored below in viral particles, these proteins are probably derived from the full‐length protein by partial degradation of regions comprising the CT.^[^ ^37^ ^]^

Next, we studied if this interaction is maintained in released viral particles. For this purpose, the culture supernatant of virus‐producing cells was collected, irradiated to induce lipid–protein cross‐linking, and viral particles were purified on OptiPrep velocity gradients. Next, photoactivatable lipid incorporation into the viral particles was quantified by a scintillation counter, and [^3^H]‐photochol was found to constitute 0.13% of the total chol molecules in these viral particles (see Supporting Experimental Section, Supporting Information for details). To study interaction, gp41 and the cross‐linked chol were immunoprecipitated from viral particles and detected by Western blot and autoradiography, respectively (Figure 1b). The results revealed that gp41—whose identity was confirmed by proteomic analysis (Figure S2, Supporting Information)—maintained the direct interaction with chol in extracellular viral particles. In addition to gp41, another protein of ≈20 kDa recognized by the anti‐gp41 antibody was found to also interact with chol. Previous studies have identified an ≈20 kDa gp41‐derived protein to comprise a fragment of the gp41 CT (Tr‐Env‐CT).^[^ ^37^ ^]^ To determine whether the chol‐bound protein in the current study corresponds to this 20 kDa protein, the corresponding band was submitted to proteomic analysis. This experiment confirmed that the chol‐bound 20 kDa protein corresponded to Tr‐Env‐CT, lacking the membrane spanning region and the ectodomain (Figure S2, Supporting Information). Interestingly, analysis of the relative chol/protein signal of Tr‐Env‐CT revealed no significant differences when compared to full‐length gp41 (Figure 1c), suggesting that the chol‐interacting region in gp41 is located in the CT of the protein. Of note, the 25 kDa protein band observed in the Western blot above Tr‐Env‐CT (Figure 1b, Ab LC) was identified by proteomic analysis to correspond to the light chain of the antibody used for immunoprecipitation, and not to be derived from gp41 (data not shown).

### 2.2 Truncation of Residues 751–854 in Gp41 Cytoplasmic Tail Inhibits Protein Interaction with Chol

Following the discovery that Tr‐Env‐CT interacts with chol at the same level as the full‐length protein, we next investigated the involvement of the gp41 CT in the interaction. Specifically, we focused on the role that the three amphipathic *α*‐helixes in the CT (known as lentiviral lytic peptides or LLP) may play in the interaction, as they have been described to 1) have membrane‐binding properties^[^ ^38^, ^39^, ^40^ ^]^ and 2) to be partially embedded in the membrane.^[^ ^41^, ^42^, ^43^ ^]^

**Figure 2:**
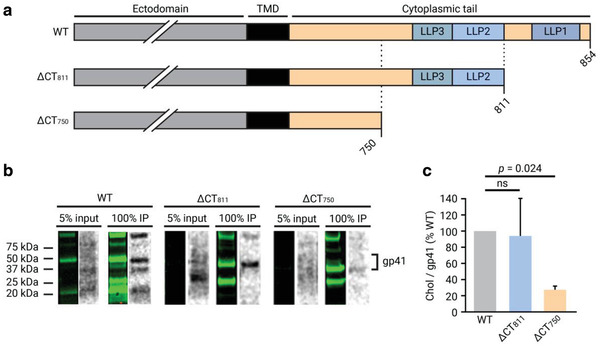
Effect of partial CT truncation in gp41 interaction with chol in purified mature viral particles. a) Schematic illustration of gp41 CT truncation mutants. The ΔCT_811_ variant contains a L812: stop codon substitution and lacks the last 43 amino‐acid residues of the cytoplasmic tail. The ΔCT_750_ variant contains a L751: stop codon substitution and lacks the last 104 amino‐acid residues of the cytoplasmic tail. b) Gp41 from input (5% of lysed viral particles) and immunoprecipitation (IP) from viral particles were detected by Western blot with the anti‐gp41 chessie‐8 antibody (green lane), and the interacting radioactively labeled lipid by autoradiography of the same membrane (gray lane). c) Quantification of the chol/protein signal ratio of the truncated gp41 proteins ΔCT_811_ and ΔCT_750_ compared to the wild‐type gp41. The bars and whiskers represent the mean ± SD of experiments from three independent viral purifications (*n* = 3). Statistical significance was assessed by analysis of variance and Tukey test.

For this purpose, two truncation variants of gp41 were constructed, 1) ΔCT_811_, with a stop codon at residue 812, lacking the C‐terminal 43 amino‐acid residues of the CT including LLP1; and 2) ΔCT_750_, with a stop codon at residue 751, lacking the C‐terminal 104 amino‐acid residues, including all three LLP sequences and 14 residues upstream (**Figure** **2**a, Table S1, Supporting Information). Proviral pCHIV plasmids containing these truncations were used to produce and purify viral particles, and interaction between gp41 and chol was studied as described above. Comparison of the Western blot and autoradiography signals (Figure 2b), and quantification of the relative chol/gp41 signals of the ΔCT_811_ and ΔCT_750_ truncation variants compared to the wild‐type protein revealed that truncation of the last 104 amino‐acid residues (ΔCT_750_) induces a loss of direct chol interaction by >70%. This loss of interaction was not due to a defect in protein or lipid incorporation into the virus particles, as demonstrated by Western blot (Figure S3, Supporting Information) and lipidomic analysis (Figure S4, Supporting Information) of purified viral particles, respectively. Truncation of only the C‐terminal 43 amino‐acid residues (ΔCT_811_) did not affect gp41–chol interaction (Figure 2c), indicating that LLP1 is not required for chol binding. These results revealed that the gp41 CT and specifically the region comprising the LLP *α*‐helixes is required for the interaction of gp41 with chol; since LLP1 is not required, it appears most likely that LLP2 and/or LLP3 are the primary determinants for this interaction.

Next, we investigated whether the cholesterol recognition amino acid consensus (CRAC) domain within the membrane proximal external region (MPER) of gp41, which has been shown to interact with chol in different in vitro studies^[^ ^44^, ^45^, ^46^, ^47^ ^]^ played any role in the interaction of gp41 with chol. For this purpose, the “LWYIK” CRAC domain in the full‐length gp41 was disrupted by introducing a conservative mutation (L677I) that has been described to hamper interaction of CRAC‐derived peptides with chol, and to cause an inhibition of fusion in cells expressing the gp41 CRAC mutant.^[^ ^48^ ^]^ Viral particles containing this mutation were purified, and chol interaction was studied. Disruption of the CRAC domain was not found to have an effect on the interaction of gp41 with chol (Figure S5, Supporting Information), excluding the CRAC domain in MPER as the region responsible for the interaction in viral particles in the steady state.

### 2.3 Gp41 Palmitoylation in the Cytoplasmic Tail Is Not Necessary for Chol Interaction

Gp41 has been described to be palmitoylated on up to two cysteine residues in the CT, and this palmitoylation has been suggested to drive Env targeting to DRMs.^[^ ^32^, ^33^ ^]^ The truncated ΔCT_750_ gp41 variant, which does not interact with chol, lacks the C762 residue described to be palmitoylated. To establish if the loss of interaction derived from the truncation of the CT_751–854_ region is related to the lack of gp41 palmitoylation, we investigated the impact of palmitoylation on the gp41–chol interaction. For this purpose, a pCHIV variant containing a substitution of the palmitoylated cysteine residue by a serine in position 762 (C762S) was constructed (Table S1, Supporting Information).

**Figure 3:**
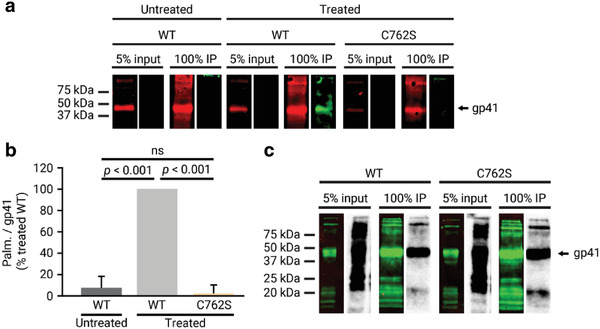
Effect of the C762S substitution on gp41 palmitoylation and gp41–chol interaction in purified mature viral particles. a) Gp41 from input (5% of lysed viral particles) and immunoprecipitation (IP) from viral particles treated or not with the clickable palmitoyl analogue precursor 15‐hexadecynoic acid were detected by Western blot with the anti‐gp41 chessie‐8 antibody (red), and the palmitoyl group of the protein was detected by the covalent binding of a IRDye800 azide fluorescent probe mediated by a click‐chemistry reaction (green). b) Quantification of the palmitoyl/protein signal ratio of the mutated gp41 protein C762S compared to the wild‐type gp41 from either treated or untreated viral particles. The bars and whiskers represent the mean ± SD of experiments from three independent viral purifications (*n* = 3). Statistical significance was assessed by analysis of variance and Tukey test. c) Gp41 from input (5% of lysed viral particles) and immunoprecipitation (IP) from viral particles were detected by Western blot with the anti‐gp41 chessie‐8 antibody (green lane), and the interacting radioactively labeled lipid by autoradiography of the same membrane (gray lane).

First, we determined the effect of the C762S substitution on gp41 palmitoylation by use of an alkyne‐modified clickable palmitoyl analogue, 15‐hexadecynoic acid. This fatty acid was fed to virus‐producing cells to become incorporated into cellular lipid metabolism and thus be a substrate for gp41 palmitoylation. Viral particles were purified from treated virus‐producing cells and gp41 was immunoprecipitated. Gp41 palmitoylation was detected by copper‐dependent click chemistry. Western blot analysis showed that wild‐type gp41 protein was efficiently palmitoylated, while the gp41 C762S variant completely lost palmitoylation (**Figure** **3**a, protein signal and palmitoylation in red and green, respectively). Quantification of the relative palmitoyl/gp41 signals confirmed that the C762S substitution led to a loss of gp41 palmitoylation (Figure 3b).

We next investigated whether the loss of palmitoylation had any effect on the interaction of gp41 with chol. Viral particles containing the gp41 C762S variant were purified, and interaction with chol was studied as above. Comparison of the Western blot and autoradiography signals showed that the C762S variant interacted with chol at a level equivalent to that observed for the wild‐type protein (Figure 3c), demonstrating that palmitoylation of gp41 at the C762 residue is not required for the interaction with chol. Furthermore, lack of palmitoylation of gp41 also did not have an effect on gp41 incorporation into the virus (Figure S6, Supporting Information).

### 2.4 Chol Interaction Does Not Depend on Maturation

**Figure 4:**
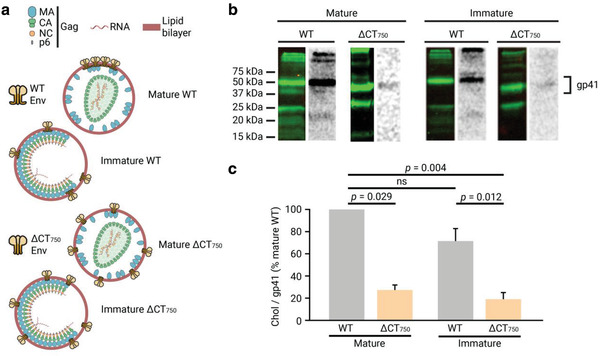
Effect of virus maturation on the CT_751–811_ dependent gp41 interaction with chol in purified viral particles. a) Schematic illustration of mature and immature viral particles containing either the full‐length wild‐type gp41 or the truncated ΔCT_750_ gp41. b) Gp41 from immunoprecipitation from mature or immature viral particles were detected by Western blot with the anti‐gp41 chessie‐8 antibody (green lane), and the interacting radioactively labeled lipid by autoradiography of the same membrane (gray lane). c) Quantification of the chol/protein signal ratio of the wild‐type and truncated ΔCT_750_ gp41 proteins in mature and immature viral particles. The bars and whiskers represent the mean ± SD of experiments from three independent viral purifications (*n* = 3). Statistical significance was assessed by analysis of variance and Tukey test.

Virus maturation is a process concomitant with budding of the viral particle from the producing cell and governs Env mobility in the membrane^[^ ^49^ ^]^ and Env clustering.^[^ ^8^ ^]^ To establish whether the maturation state of the virus has an effect on gp41 association with chol, we investigated whether this interaction is also observed in immature viral particles containing a mutation in the viral protease. Mature and immature particles containing either wild‐type or truncated (ΔCT_750_) gp41 (**Figure** **4**a) were purified, and chol interaction was studied by Western blot and autoradiography (Figure 4b). Quantification of the relative chol/gp41 signal (Figure 4c) revealed that the maturation state of the virus did not have an effect on gp41–chol interaction. Wild‐type gp41 interacted with chol at the same level in mature and immature particles, and this interaction was lost in both cases when the CT was truncated. This loss of interaction was not caused by differences in protein incorporation into the virus (Figure S7, Supporting Information).

### 2.5 Gp41 Cytoplasmic Tail and Chol Govern Envelope Clustering

**Figure 5:**
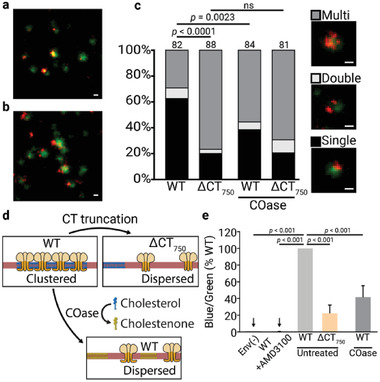
Env distribution patterns of virus particles with full‐length or the truncated ΔCT_750_ gp41 and its effect on entry. a,b) Representative images of Vpr.eGFP (green) and Env (orange) signal of virus particles with full‐length and the truncated ΔCT_750_ gp41, respectively. c) Env distribution patterns of virus particles with full‐length and the truncated ΔCT_750_ gp41, with and without Cholesterol oxidase (COase) treatments. The number of viral particles quantified in each preparation is represented above the bars (*n* > 80). The statistical significance was assessed by *χ* ^2^ test for independence at two degrees of freedom. Images show examples of virus particles for each Env distribution class. Scale bars: 100 nm. d) Schematic representation of Env cluster dispersion caused by gp41 ΔCT_750_ truncation, and by conversion of chol to cholestenone by COase. Viral membrane in red, Env trimers in orange, chol molecules in blue, and cholestenone molecules in green. e) Effect of ΔCT_750_ truncation and COase treatment in entry capacity of concentrated BlaM.Vpr mature viral particles. For each variant, a viral amount corresponding to 30 ng of CA was incubated with Jurkat E6‐1 cells. As a control, viral particles lacking Env (Env(−)), and WT viral particles treated with the entry inhibitor AMD3100 (WT+AMD3100) were used. COase‐treated viral particles were incubated with 0.5 U of COase for 30 min at 37 °C. Untreated viral particles were incubated for 30 min at 37 °C without enzyme. The relative fluorescent intensity ratios of the cleaved (blue) and uncleaved (green) Vpr.BlaM fluorescent substrate CCF2 loaded into the cells was measured for each variant. The bars and whiskers represent the mean ± SD of experiments from five independent viral purifications (*n* = 5) with four replicas each. Statistical significance was assessed by analysis of variance and Tukey test.

Although maturation dependent Env clustering has been shown to be necessary for efficient viral entry of wild‐type HIV‐1 and the gp41 CT is involved in this process,^[^ ^8^ ^]^ the mechanism that governs Env clustering and the specific role of the gp41 CT in this process has yet to be elucidated. Given the importance of chol for viral infectivity and its binding to the gp41 CT, we wanted to determine whether the gp41–chol interaction could be the underlying mechanism governing Env clustering. To this end, the effect of the ΔCT_750_ truncation and of chol interaction on Env clustering was studied by analyzing Env distribution in viral particles using STED super‐resolution nanoscopy. Individual viral particles were identified by pseudotyping with the viral accessory protein Vpr tagged with enhanced green fluorescent protein (Vpr.eGFP). To study Env clustering, viral particles containing either wild‐type or truncated ΔCT_750_ gp41 variants were immunolabeled against Env (**Figure** **5**a,b). Acquisition of Env signal (red) and Vpr.eGFP signal (green) confirmed that the ΔCT_750_ truncation induced a shift toward a dispersed distribution and loss of clustering when compared to the wild‐type protein (Figure 5c), as reported for another CT truncation in a previous study.^[^ ^8^ ^]^ Immature viral particles exhibited a broadly distributed Env pattern independently of the gp41 variant as reported before^[^ ^8^ ^]^ (Figure S8, Supporting Information), which may be explained by the sequestering of Env by the underlying rigid Gag lattice in immature viral particles.^[^ ^49^, ^50^ ^]^ Of note, ≈20% of the immature viral particles, as well as the truncated ΔCT_750_ gp41 variant, were categorized as single focus particles. We expect this residual clustering to be derived in part from a resolution limit in the STED clustering assay, which cannot differentiate Env distribution to the molecular level. This residual clustering (≈20%) would thus represent a lack of sensitivity in the assay to resolve Env molecules that are dispersed, but separated by a distance (≈10–20 nm) lower than the resolution limit of the technique (≈40 nm).

To determine whether the gp41 CT interaction with chol is required for Env clustering, we investigated the effect of chol depletion on Env distribution in the viral membrane. For this purpose, Vpr.eGFP viral particles immunolabeled against Env were treated with cholesterol oxidase (COase). In contrast to other chol depleting agents such as *β*‐cyclodextrin, COase does not remove chol molecules from the membrane and thus does not change the total lipid molecules in the virus. This enzyme converts up to 50% of the viral membrane chol into cholestenone,^[^ ^51^ ^]^ removing the polar hydroxy chol headgroup that is known to interact with proteins.^[^ ^52^ ^]^ The headgroup was previously shown to also be important for the self‐aggregation of HIV‐1 gp41‐derived peptides in the presence of chol‐rich model membranes.^[^ ^53^ ^]^ Treatment with COase of wild‐type viral particles carrying the already clustered Env proteins revealed that partial conversion of chol to cholestenone caused dispersion of the clustered Env. In contrast, no significant additional effect was observed regarding the distribution of the already disperse truncated ΔCT_750_ gp41 variant (Figure 5c). These results indicated that the interaction between the CT_751–854_ region of gp41 and chol is necessary for efficient Env clustering, and depletion of chol from the viral membrane disaggregates the already created Env clusters.

We next investigated whether the loss of Env clustering also resulted in a loss of entry capacity. This was analyzed using the BlaM assay,^[^ ^54^ ^]^ which detects cytoplasmic entry of a modified *β*‐lactamase incorporated into the virion by cleavage of a BlaM substrate. For this purpose, viral particles carrying Vpr fused with the *β*‐lactamase enzyme (Vpr.BlaM) were produced. Comparative entry experiments were performed on Jurkat E6‐1 cells for particles carrying wild‐type gp41 or the ΔCT_750_ gp41 variant and treated or not with COase. After loading with the *β*‐lactamase fluorescent substrate CCF2, the entry capacity of the viral particles was calculated as a relative fluorescent intensity ratio of the cleaved (blue) and uncleaved (green) fluorescent substrate. Figure 5d shows that the ΔCT_750_ truncation or partial conversion of chol to cholestenone in the viral membrane by the COase resulted in a significant decrease (>70% and ≈60%, respectively) in viral entry capacity, indicating that inhibition of the gp41–chol interaction hampers HIV‐1 entry, in accordance with the observed loss of Env clustering.

## 3 Discussion

Upon maturation, HIV‐1 Env trimers redistribute into a single cluster, facilitating fusion with the host cell.^[^ ^8^ ^]^ Although several factors seem to regulate this clustering, little is known about the molecular mechanisms governing this process. Chol, one of the main lipids in HIV‐1, is also a key requirement for infectivity, but the molecular mechanism explaining its precise role in the entry process is currently not well understood. Here, we have studied the interaction of gp41 with chol in the viral membrane and the role of this interaction for the redistribution of Env upon maturation.

The gp41 CT is unusually long when compared to the related retroviruses and the conserved sequences in it suggest it to be a key component of the protein. During viral morphogenesis, the gp41 CT is important for incorporation of the protein into budding sites, either by direct interaction with the underlying Gag matrix^[^ ^55^, ^56^, ^57^ ^]^ or by its preferential partitioning into specific lipid nanodomains created or selected by Gag.^[^ ^58^ ^]^ Once incorporated into the virus, the gp41 CT also regulates protein activity and distribution.^[^ ^8^ ^]^ Additionally, the gp41CT has been postulated to be involved in the interaction with chol in the viral membrane based on the partitioning of Env into DRMs,^[^ ^34^ ^]^ but direct interaction of gp41 with chol in the native viral membrane has not been observed so far.

Using a well‐established photoaffinity strategy with a bifunctional photoactivatable chol analogue, we have proven that gp41 directly interacts with chol both in virus‐producing cells and in released purified viral particles. The photoactivatable [^3^H]‐photochol incorporated into viral particles was found to constitute only around 0.13% of the total chol molecules in the virus, which supports the notion that cross‐linking of the lipid with gp41 detects specific interaction with chol, and is not an artifact due to the high chol amount (≈50%) in the virus membrane. Additionally, we found that this interaction is mediated by the gp41 CT, and a peptide corresponding only to the CT was also cross‐linked to chol within viral particles. We further demonstrated that the region comprising residues 751–854 in the gp41 CT, and in particular the region containing LLP2 and 3, is responsible for the interaction, while LLP1 appears to be dispensable. All three LLP *α*‐helixes have membrane‐binding properties^[^ ^38^, ^39^, ^40^ ^]^ and are partially embedded in the inner leaflet of the viral membrane,^[^ ^41^, ^42^, ^43^ ^]^ which supports their role in the interaction of gp41 with chol. Furthermore, previously published work has shown that selective pressure results in HIV‐1 partially overcoming the antiretroviral effect of a chol‐binding compound, amphotericin B methyl ester (AME), by developing truncations in the gp41 CT. This AME resistant mutant is generated by cleavage of the last 104 amino‐acid residues in gp41 CT—equivalent to the ΔCT_750_ gp41 variant in our work—by the viral protease,^[^ ^59^ ^]^ strongly suggesting a relationship between gp41 CT and chol. Indeed, the results obtained in our work could provide a more in depth explanation of this effect. When AME binds and sequesters chol in the viral membrane, the Env trimers interacting with chol via the gp41 CT_751–854_ region are also sequestered by extension, which hampers their activity and function. Truncation of the gp41 CT_751–854_ would in contrast uncouple the Env trimers from the chol molecules sequestered by AME, allowing their diffusion. As explained below, this CT truncated viral particles could retain fusion activity in certain cell types by an increased incorporation of Env into the virion—partially overcoming the necessity to cluster, by a receptor‐induced Env clustering, or by a combination of both.

Interestingly, the canonical chol‐binding CRAC domain located in the gp41 MPER, previously shown to bind chol molecules in in vitro studies,^[^ ^44^, ^45^, ^46^, ^47^ ^]^ was not found to be required for gp41–chol interaction in mature viral particles in the steady state. One possible explanation for this discrepancy relies on the fact that these reports studied the interaction of CRAC with lipids using indirect in vitro techniques such as localization of Env to DRMs,^[^ ^45^ ^]^ binding of CRAC‐derived peptides to membranes,^[^ ^47^ ^]^ binding of peptides to agarose‐immobilized chol derivatives,^[^ ^44^ ^]^ or antibody binding assays with CRAC mutated gp41 proteins.^[^ ^46^ ^]^ None of these techniques provide absolute proof of the association of gp41 with chol or chol‐enriched nanodomains, nor of the specific role of the CRAC domain in said interaction, in functional viral particles. The observed differences could additionally be explained by the fact that our study was carried out with free viral particles, where Env was not engaged to cellular receptors. It cannot be excluded that the CRAC sequence could play a role in protein–lipid interaction in other protein conformations, such as during Env trafficking in viral morphogenesis or when Env is in an intermediate fusion state.

Since chol and chol‐rich nanodomains are important regulators of the function and localization in the membrane of several proteins,^[^ ^28^, ^29^, ^30^ ^]^ we next tried to pinpoint the physiological relevance of the gp41–chol interaction in HIV‐1. Using Env localization analysis by STED nanoscopy, we determined that truncation of the chol‐interacting CT_751–854_ region inhibited clustering of Env inducing a dispersed phenotype. Previous reports have suggested that a cross‐talk between gp41 CT and the underlying MA lattice,^[^ ^55^, ^60^ ^]^ or interaction between trimers via the CT^[^ ^8^ ^]^ could be the driving force behind the redistribution of Env, but here we demonstrate that the already clustered wild‐type Env acquires a dispersed phenotype after chol depletion caused by the treatment with COase. A general assumption is that chol is able to fluidize membranes composed of lipids with long saturated chains, such as those present in HIV‐1, so conversion of chol to cholestenone by COase should lead to an increased rigidity of the HIV‐1 membrane. Even with this rigidification and the derived decreased protein mobility in the membrane,^[^ ^49^ ^]^ the already clustered wild‐type Env trimers disperse after the COase treatment, further supporting the suggestion that the specific interaction of chol with gp41 is critical for clustering while gp41 CT–MA or CT–CT interactions alone, which should be intact in the COase‐treated wild‐type virus, are not sufficient.

Viral entry assays showed that inhibition of Env clustering, either by truncation of the CT or depletion of chol, resulted in decreased entry presumably by hindering the coordinated action of Env trimers, as reported before.^[^ ^8^ ^]^ Interestingly, as introduced above, previous studies found that truncation of gp41 CT can render fully infectious viral particles in certain cell types,^[^ ^57^, ^59^ ^]^ which seems to be coupled with an increased incorporation of Env into the virus.^[^ ^8^, ^57^ ^]^ Env clustering is suggested to be an evolutionary mechanism to overcome the low number of Env trimers in the virus, so retention of infectivity in certain cell types after CT truncation may be explained by the increased number of incorporated Env trimers induced by the mutation. This could in turn permit the coordinated action of several neighboring trimers without the need to distribute them into a cluster. Nevertheless, this effect is highly cell‐type dependent and is only observed in some appropriately called “permissive” cells, while “non‐permissive” lymphocytes such as the Jurkat cells used in our work, among others, require the gp41 CT for infectivity.^[^ ^57^, ^61^ ^]^ Similarly, the residual fusion observed in the truncated gp41 variant can be explained by the fact that Env dispersion caused by complete CT truncation of gp41 has previously been shown to be partially rescued after engagement with cellular receptors.^[^ ^8^ ^]^ This is probably due to retention of Env trimers once they have engaged with CD4 receptors, ultimately presenting a receptor‐induced clustered phenotype. This would be facilitated by the increased mobility of Env in the viral membrane after virus maturation^[^ ^49^ ^]^ and by the increased incorporation of the truncated Env into the viral particles. This receptor‐induced clustering would allow the virus to retain residual fusion capabilities after engagement to the host cell.

Our findings demonstrate that Env clustering requires the interaction of gp41 with chol in the viral membrane, which could explain the mechanism of action of several antiretroviral drugs that deplete^[^ ^22^, ^23^, ^24^, ^25^ ^]^ or sequester^[^ ^26^, ^27^ ^]^ chol, or target the structure and properties of raft‐like lipid nanodomains^[^ ^20^ ^]^ by providing a link between chol in the viral membrane and viral entry. Additionally, our findings come in excellent agreement with and provide further insight to a recent study that demonstrated that the entry inhibitory effect of SERINC5, a host cell protein incorporated into the virus, is based in its disruption of Env clusters. Based on the observation that SERINC5 did not codistribute with Env the authors conclude that its effect in clustering is indirect, and hypothesize that its effect could be based on sequestering of viral membrane chol by SERINC5.^[^ ^62^ ^]^ Our results directly support the hypothesis that sequestering of chol by SERINC5 would remove the molecular switch driving Env redistribution and hamper entry.

We anticipate that the approach followed in our work could shed new light into how protein–chol interactions in the viral membrane could regulate glycoproteins functions in other viruses. In influenza, the fusion protein hemagglutinin also seems to interact with chol,^[^ ^63^ ^]^ and modulation of chol‐mediated membrane properties has been found to disrupt infectivity in the virus,^[^ ^64^ ^]^ suggesting that a direct interaction with chol could also regulate protein function and infectivity in this virus. Chol has also been shown to be a key component of viral infectivity in Ebola virus, where it tightly regulates assembly of new viral particles in the plasma membrane by targeting the viral GP proteins to chol‐rich nanodomains^[^ ^65^ ^]^ and recently, in SARS‐CoV‐2, it has been found to be an important requisite for infectivity by inducing trafficking of ACE2 to viral endocytic entry points, as well as increasing SARS‐CoV‐2 binding to the cells.^[^ ^66^ ^]^

## 4 Conclusion

**Figure 6:**
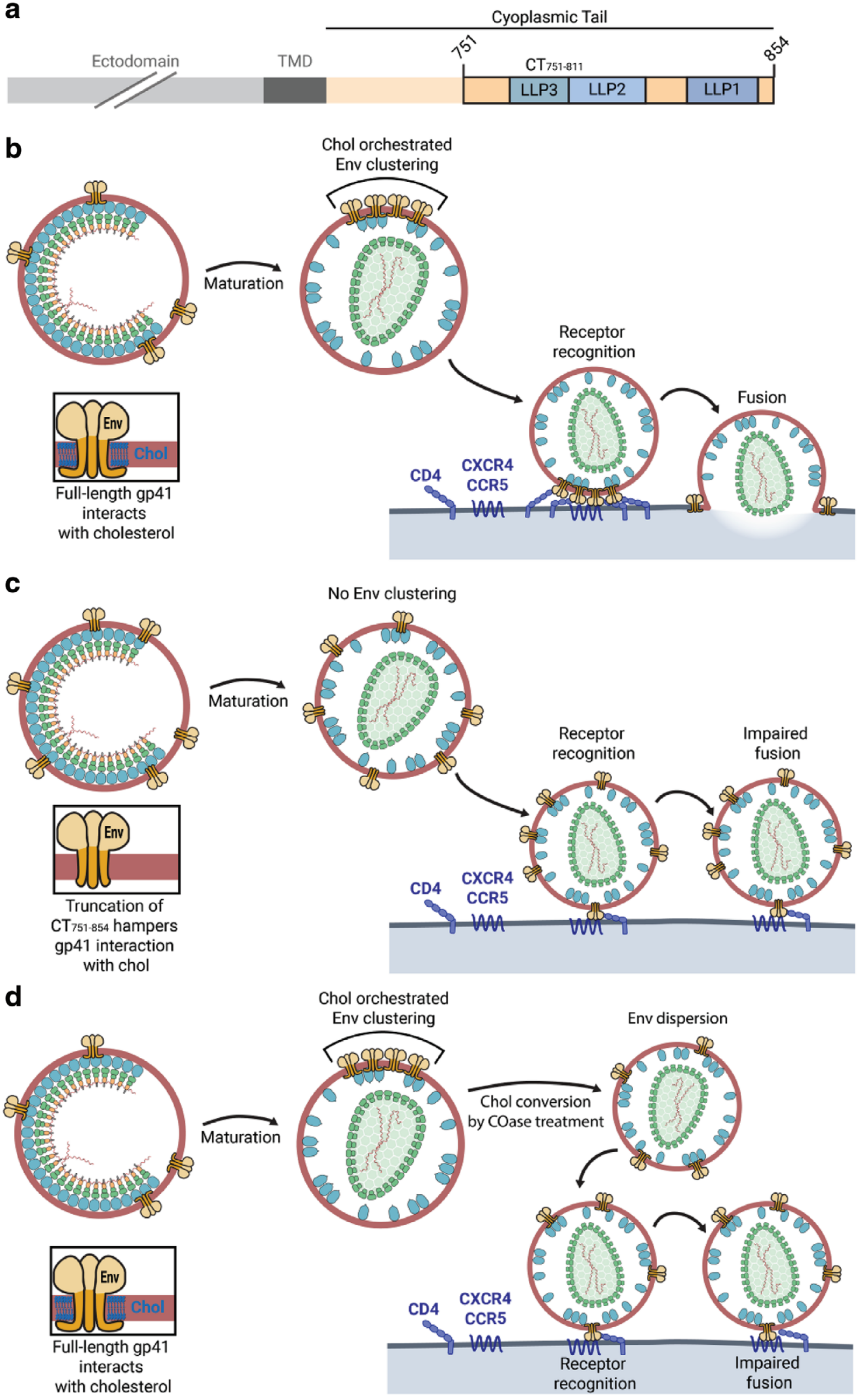
Env clustering depends on CT_751–811_ mediated chol interaction and maturation state. a) Schematic illustration of the CT_751–854_ region responsible for gp41–chol interaction. b) In wild‐type HIV‐1 particles, the full‐length gp41 protein interacts with viral membrane chol via the CT_751–854_ region. Upon maturation, Env trimers are free to diffuse and coalesce into a single focus mediated by their interaction with chol. This clustering enables efficient fusion with the host cell. c) When the CT_751–854_ region is truncated, the protein loses the capacity to interact with chol. Upon maturation, truncated Env trimers cannot coalesce into a single focus as they are excluded from chol‐rich domains. The absence of an Env cluster results in a defect in fusion with the host cell. d) Disruption of the gp41–chol interaction by treatment of wild‐type HIV‐1 particles with COase and conversion of chol to cholestenone, induces a dispersion of the already clustered Env particles, resulting in a phenotype similar to the truncated Env trimers.

Collectively, our results reported here support a maturation dependent Env clustering mechanism governed by the interaction of gp41 with chol in the viral membrane via the CT_751–854_ region (**Figure** **6**a). In an immature state, the underlying rigid Gag lattice sequesters Env trimers in place, not allowing their diffusion and redistribution. Upon virus maturation and Env release from the Gag lattice, the now‐free‐to‐diffuse Env trimers^[^ ^49^ ^]^ associate into a cluster driven by their lateral self‐aggregation induced by their interaction with chol. This clustering facilitates an efficient fusion with the host cell, viral entry, and infectivity (Figure 6b). Since the interaction between gp41 and chol is mediated by residues 751–854 in the CT, truncation of this region induces a loss of interacting capacity with chol. Upon maturation and increase of Env mobility, because the truncated gp41 cannot interact with chol, it does not coalesce into a single cluster, remaining dispersed in the viral membrane. If the number of Env molecules in the virus remains low, dispersion of Env hampers or delays coordinated action of several trimers, decreasing viral and host cell membrane fusion and entry (Figure 6c). Similarly, depletion of chol from the viral membrane removes the driving force behind Env clustering, inducing a dispersion of the already clustered wild‐type gp41 and thus also inhibiting clustering and subsequent entry (Figure 6d).

This chol dependent clustering could be explained by several related molecular mechanisms. After virus maturation, the interaction of gp41 with chol could drive its partitioning into specific lipid nanodomains, inducing a clustering of Env trimers. Alternatively, interaction of gp41 with chol could act as a nucleation site for the generation of new protein–lipid nanodomains to which neighboring Env trimers and chol molecules are recruited. Due to the small size of the viral particles and even smaller size of protein–lipid nanodomains, elucidating the specific molecular mechanism would necessitate super‐resolution fluorescence nanoscopy techniques with much higher spatiotemporal resolution capabilities.

## 5 Experimental Section

##### Experimental Design

The main goal of this work was to unravel whether interaction of gp41 with chol was the driving force governing Env clustering. To accomplish this, the authors studied and demonstrated the direct interaction of gp41 in viral particles with viral membrane chol by using a functionalized photoactivatable chol analogue. The authors then further determined the region involved in the interaction by truncation of the CT of gp41 and mutations in several other regions of interest. To determine whether the gp41–chol interaction was the driving force behind Env clustering, the effect of the disruption of this interaction in the distribution of Env in the virus was analyzed using super‐resolution STED nanoscopy. The authors finally established the biological outcome of Env clustering studying the entry capacity of the viral particles using a BlaM assay.

##### Plasmids and Cells

Non‐infectious HIV‐1 particles were producing using the pCHIV plasmid,^[^ ^67^ ^]^ which expressed all of the HIV‐1_NL4‐3_ proteins except Nef, and lacks the long‐terminal repeat sequences necessary for competent replication. pCHIV derivatives were used for studying the effect of gp41 mutations, and maturation state of the virus. For cellular interaction studies with only the Env protein, HIV‐1_NL4‐3_ Env expressing pCAGGS construct was used. Viral particles pseudotyped with BlaM.Vpr for entry capacity studies were produced by cotransfection with pMM310 plasmid. Viral particles pseudotyped with eGFP.Vpr for Env distribution studies were produced by cotransfection with eGFP.Vpr plasmid. Detailed information on all of the constructs is listed in Table S1, Supporting Information.

293T cells were maintained at 37 °C and 5% CO_2_ in DMEM GlutaMAX High Glucose culture medium supplemented with 10% FBS, and 100 U mL^−1^ Penicillin–Streptomycin. Jurkat E6‐1 cells were maintained at 37 °C and 5% CO_2_ in RPMI 1640 GlutaMAX culture medium supplemented with 10% FBS, and 100 U mL^−1^ Penicillin–Streptomycin.

##### Viral Particle Purification

293T at 60% confluency were transfected with the proviral pCHIV plasmid by calcium phosphate precipitation. Viral particle purification was carried out as described before.^[^ ^20^, ^68^, ^69^ ^]^ Briefly, culture medium was harvested, cleared by filtration, and viral particles were concentrated by ultracentrifugation through a cushion of 20% w/v sucrose. Pelleted HIV‐1 particles were resuspended and further purified by velocity gradient purification using an OptiPrep gradient (Axis‐Shield). The visible virus fraction was collected, pelleted by ultracentrifugation, resuspended in 150 mm NaCl, 10 mm Hepes pH 7.4, aliquoted, frozen in liquid nitrogen, and stored at −80 °C. Virus particle concentration was determined by an anti‐CA Western blot.

##### Gp41–Chol Interaction Assay by Photoaffinity Labeling

For gp41–chol interaction studies 293T cells were transfected in 10 cm dishes with either an Env expressing pCAGGS plasmid (4 µg/dish) or a proviral pCHIV plasmid (10 µg/dish) as described before. 16 h after, transfection cells were incubated with 100 µCi/dish of [^3^H]‐photochol diluted in 10 mL/dish of culture medium supplemented with 10% delipidated FBS for 24 h. Then, the culture medium containing the viral particles was collected. Cells were washed with PBS, and irradiated with UV light (360 nm) in ice cold PBS for 5 min to induce cross‐linking; afterwards cells were scraped, pelleted, and lysed with a modified RIPA buffer (20 mm HEPES, 100 mm NaCl, 5 mm EDTA, 0.5% w/v sodium deoxycholate) for 1 h at 4 °C. The culture medium containing viral particles was cleared by filtration, irradiated with UV light for 5 min at 4 °C to induce cross‐linking, and viral particles were purified as before. After purification yield quantification, viral particles equivalent to 1 µg of CA were lysed with modified RIPA buffer for 1 h at 4 °C. 5% of the total volume of lysed cells or viral particles was stored for use as an input in Western blot and autoradiography studies.

Gp41 was immunoprecipitated from the lysed cellular and viral samples by incubation overnight at 4 °C with Protein G Sepharose 4 Fast Flow (GE Healthcare) beads covalently bound to anti‐gp41 chessie‐8 antibody. Beads were pelleted, washed, and boiled with SDS‐PAGE 6x sample buffer at 95 °C for 5 min to release the proteins from the beads. The samples were loaded into an SDS‐PAGE (12.5%) for subsequent analysis by Western blotting. After incubation of Western blot membrane with chessie‐8 mouse anti‐gp41 primary antibody (1:2000 overnight at 4 °C), membrane was developed with anti‐mouse IRDye 800 secondary antibody (1:15 000 for 45 min at RT) diluted in Odyssey Blocking Buffer (LI‐COR). Detection of the proteins was carried out using the LI‐COR Odyssey imaging system. To detect the lipid signal, the Western blot membranes were then dried and subjected to autoradiography for 18 h in a BetaIMAGER(Biospace Lab).

##### Gp41 Palmitoylation Assay

Gp41 palmitoylation was studied with the use of an alkyne‐modified clickable palmitoyl analogue, 15‐hexadecynoic acid. 16 h after, transfection 293T cells were treated or not with 500 µm of 15‐hexadecynoic acid diluted in 10 mL/dish of culture medium supplemented with 10% delipidated FBS for 24 h. Then, the culture medium containing the viral particles was collected and cleared by filtration, and viral particles were purified as before. After purification yield quantification, viral particles equivalent to 1 µg of CA were lysed with EDTA free modified RIPA buffer (20 mm HEPES, 100 mm NaCl, 0.5% w/v sodium deoxycholate) for 1 h at 4 °C. Gp41 was immunoprecipitated as before, and the palmitoyl analogue incorporated into gp41 was labeled by a copper‐dependent click‐chemistry reaction.^[^ ^70^ ^]^ Briefly, the immunoprecipitated samples bound to the beads were incubated with 0.1 mm TBTA, 1 mm Cu(III)SO_4_, 10 mm ascorbic acid, and 10 µm of IRDye800 azide fluorescent dye for 1 h at dark and RT with constant stirring. The beads were then washed and incubated at 60 °C for 5 min with 6x sample buffer containing 150 µm *β*‐mercaptoethanol and loaded into an SDS‐PAGE. A Western blot was developed with chessie‐8 mouse anti‐gp41 primary antibody (1:2000) overnight at 4 °C and anti‐mouse IRDye 800 secondary antibody (1:15 000) for 45 min at RT diluted in Odyssey Blocking Buffer (LI‐COR). Detection of the proteins and clicked palmitoyl analogue was carried out using the LI‐COR Odyssey imaging system, with the protein signal in the 680 channel (red) and the palmitoyl signal in the 800 channel (green).

##### Env Distribution Assay

For Env distribution measurements, purified HIV‐1 particles (≈1 µg of CA) were adhered to poly‐L‐lysine (Sigma)‐coated coverslips for 15 min. Coverslips were blocked using 2% bovine serum albumin (Sigma)/PBS for 15 min. Particles were stained for Env using 10 ng µL^−1^ 2G12 Fab fragments, and anti‐human Abberior STAR RED (KK114) conjugated Fab fragments for 1 h each in the blocking buffer. Following immunostaining, particles were washed in PBS, briefly fixed using 3% PFA/PBS, overlaid with SlowFade Diamond mounting medium (Thermo Fisher Scientific) and imaged using STED nanoscopy. All above steps were carried out at room temperature. For COase treatment, coverslip adhered and Env immunostained virus particles were incubated with 0.5 U COase from *Streptomyces species* (Sigma) for 30 min at 37 °C prior to washing, post‐fixing, and mounting.

Super‐resolution analysis of HIV‐1 Env distribution was performed using Leica SP8 STED 3X microscope (Mannheim, Germany) equipped with a 100 ×/1.4 NA oil immersion STED objective. 2‐color STED images of Env (Abberior STAR RED) and Vpr.eGFP signal were acquired sequentially for each channel using 637 and 498 nm lines from the white light laser. Abberior STAR RED and eGPF signal was depleted with a donut‐shaped 775 and 592 nm pulsed STED lasers, respectively.

Under these conditions, ≈40 and 100 nm lateral axial resolution (full‐width‐at‐half‐maximum) was achieved in Env (Abberior STAR RED) and eGFP.Vpr acquisition channels, respectively (estimated from fluorescent bead and single fluorescent antibody molecule measurements). STED images were acquired with following parameters: pinhole size: 1 Airy; dwell time: 1.2 µs pixel^−1^; and XY pixel size: 20 nm. Following acquisition, STED images were thresholded and smoothed (Gaussian Blur, radius = 15 nm) using Fiji (ImageJ distribution) software. Env distribution was assessed by manual classification of Env signals associated with individual virus particles into three distribution patterns: a single cluster, two clusters, and multiple clusters.^[^ ^8^ ^]^

##### Viral Entry Assay

HIV‐1 entry assay were performed as described before.^[^ ^54^ ^]^ Viral particles pseudotyped with a Vpr‐*β*‐lactamase fusion protein (BlaM.Vpr) were obtained by cotransfection of 293T cells with 10 µg of pCHIV and 1 µg of pMM310^[^ ^71^ ^]^ plasmids. Viral particles were concentrated and quantified as described before. Viral particles equivalent to 30 ng of CA were added to 10^5^ Jurkat E6‐1 cells in a 96 well plate, spinoculated by centrifugation at 1000 x *g* for 5 min and incubated for 2.5 h. For COase treatment, viral particles were incubated with 0.5 U COase from *Streptomyces species* (Sigma) for 30 min at 37 °C prior to addition to the cells. Untreated viral particles were incubated for 30 min at 37 °C without the enzyme. As negative controls of entry, viral particles lacking Env (obtained with the pCHIV Env(−) construct), and wild‐type particles treated with the entry inhibitor AMD3100 (0.5 µm)^[^ ^72^ ^]^ were used. After virus incubation, cells were washed and incubated with 60 µL/well of CCF2 *β*‐lactamase substrate loading solution (Invitrogen; prepared according to manufacturer's instructions) for 17 h at room temperature in the dark. CCF2 *β*‐lactamase substrate cleavage was measured by recording the relative fluorescence intensities of the substrate (excitation = 409 nm, emission = 528 nm) and product (excitation = 409 nm, emission = 460 nm). Background from unstained cells was subtracted, and the relative ratio of emission intensities at 460/528 nm (product/substrate) was calculated.

##### Statistical Analysis

For gp41–chol interaction, Env incorporation, lipidomic analysis, and viral entry assays data were normalized to wild‐type particle signal. In Env distribution studies by STED the number of particles with each distribution was expressed as a percentage of the total sample size. The sample size was always *n* > 3, but the specific *n* value for each experiment is detailed in the respective figure and figure legends. Experimental groups were compared and significance determined by analysis of variance and Tukey test when more than two samples were compared, and by Student's *t*‐test when two samples were compared, using SigmaPlot. Data are represented as means with standard deviation (±SD) unless otherwise stated. The statistical significance of Env distribution was assessed by *χ* ^2^ test for independence at two degrees of freedom.

## Conflict of Interest

The authors declare no conflict of interest.

## Supporting information

Supporting Information
