# Supplementary material for: Cholesterol in the Viral Membrane is a Molecular Switch Governing HIV‐1 Env Clustering
Source: Adv Sci (Weinh). 2020 Dec 21;8(3):2003468. doi: 10.1002/advs.202003468 (PMC7856888; doi:10.1002/advs.202003468)
Supplement: Supplementary file 1 — Supporting Information [file ADVS-8-2003468-s001.pdf]

© 2020 Wiley-VCH GmbH

Supporting Information

for *Adv. Sci.,* DOI: 10.1002/advs.202003468

Cholesterol in the viral membrane is a molecular switch governing HIV-1 Env clustering

Jon Ander Nieto-Garai, Aroa Arboleya, Sara Otaegi, Jakub Chojnacki,
Josefina Casas, Gemma Fabrias, F-Xabier Contreras, Hans-Georg Kräusslich,
Maier Lorizate*

Supporting Information

Cholesterol in the viral membrane is a molecular switch governing HIV-1 Env clustering

*Jon Ander Nieto-Garai, Aroa Arboleya, Sara Otaegi, Jakub Chojnacki, Josefina Casas, Gemma Fabrias, F-Xabier Contreras, Hans-Georg Kräusslich, Maier Lorizate**

Supporting Experimental Section

Synthesis of lipid analogues.

The photoactivatable chol analogue (photochol) was synthesized by conversion of 6-keto-5α-cholestan-3β-ol (Sigma) to 6-azi-5α-cholestan-3β-ol, and tritium-labeled to obtain [^{3}H]-photochol ([3α-^{3}H]6-azi-5α-cholestan-3β-ol) by reduction using [^{3}H]$NaBH_4$ as described before[1].

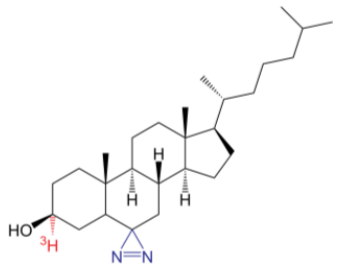

The palmitic acid analogue 15-Hexadecynoic acid was synthesized as described before[2] from commercial 16-bromohexadecanoic acid by pyridinium chlorochromate oxidation, Bestmann-Ohira reaction and ester cleavage.

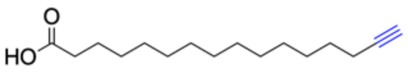

Lipid incorporation.

Lipid incorporation yield was studied in HEK 293T seeded in 35 mm dishes. 10 µCi/dish of [^{3}H]-photochol was added in 2 mL DMEM GlutaMAX^{TM} High Glucose culture medium supplemented with 10% delipidated or lipidated FBS, and 100 U/ml Penicillin-Streptomycin, and incubated with the cells for different times. Cells were then scraped, pelleted, and resuspended in 100 µL of methanol and 10 µL of water, and vortexed vigorously. The samples were centrifuged at 14,500 rpm for 15 min to separate the aqueous and organic phases. The upper organic phase containing the extracted lipids was collected, and radioactivity of the sample (in disintegrations per minute or DPM) was determined by a Tri-Carb 2900TR (Perkin Elmer) scintilliation counter. The radioactive signal in µCi was obtained from the DPM data with the following formula:

$$1\ \mu Ci = 2.2 \times 10^6\ DPM$$

To determine the lipid incorporation into the virus, viral particles were purified by Optiprep gradient and their lipids were extracted as explained above and the radioactivity of the sample was determined by a scintilliation counter. To calculate the number of [^{3}H]-photochol in each viral particle, the CA amount of the sample was quantified by an anti-CA Western blot, and assuming ~2500 molecules of CA[3,4] and ~80,000 molecules of chol[4,5] in each viral particle, the percentage of incorporated [^{3}H]-photochol compared to total chol was calculated.

Chessie-8 coupling to beads.

For gp41 immunoprecipitation the anti-gp41 chessie-8 antibody was covalently coupled to Protein G Sepharose 4 Fast Flow (GE Healthcare) beads, so that the heavy chain of that the antibody, with a similar size to gp41, does not interfere in the Western blot detection of the protein. 50 µl aliquots of washed Protein G Sepharose beads were incubated with 10 µg of chessie-8 antibody overnight at 4 ºC and constant stirring. The beads were washed twice and resuspended in 1 mL DPM borat buffer (0.2M $Na_2B_4O_7$, 0.2M H_3BO_3, 5.184 mg/ml dymethyl

pimelimidate dyhydrochloride) and incubated for 30 min at RT and constant stirring. The beads were washed twice and resuspended in 1 mL 0.2M ethanolamine pH 8.0 buffer and incubated for 2 h at RT and constant stirring. Finally, the beads were extensively washed with PBS before their use.

Quantification of gp41 incorporation into viral particles.

gp41 incorporation into viral particles was quantified by Western blot analysis of gp41 content. Amounts of purified viral particles corresponding to 120, 100, 50 and 25 ng of CA were loaded into a SDS-PAGE and a Western blot was developed with chessie-8 mouse anti-gp41 primary antibody (1:2000) overnight at 4 ºC and anti-mouse IRDye 800 secondary antibody (1:15,000) for 45 min at RT diluted in Odyssey Blocking Buffer (LI-COR). Detection of the proteins was carried out using the LI-COR Odyssey imaging system. The intensity signal of the protein band corresponding to gp41 was plotted against the loaded ng of CA, and a linear regression curve was obtained. The slopes of the linear regression curves of the variants (corresponding to the gp41/CA ratio) were compared to the wild-type particles to calculate the relative gp4 content of the viral particles.

Lipidomic analysis.

Lipids were extracted with a methanol-chloroform (1:2, vol/vol) solution containing internal standards (N-dodecanoylsphingosylphosphorylcholine, 16:0 D31_18:1 phosphocholine, 0.2 nmol each, and 2 nmol of stigmasterol from Avanti Polar Lipids). Extracts were evaporated and solved in MeOH before analysis. Lipids were analysed by liquid chromatography- high resolution mass spectrometry using an Acquity UHPLC BEH C8 column and an Acquity ultra high-performance liquid chromatography (UHPLC) system (Waters, USA) connected to a Time of Flight (LCT Premier XE) Detector. Positive identification of compounds was based

on the accurate mass measurement with an error B5 ppm and its LC retention time, compared with that of a standard (92%).

Proteomic analysis.

Proteins detected in the gp41 immunoprecipitation were subjected to proteomic analysis by mass spectrometry. gp41 was immunoprecipitated as before from purified viral particles and loaded into an SDS-PAGE. The gel was then stained with Coomassue blue, and the bands of an apparent ~41 kDa (gp41), ~20 kDa (Tr-Env-CT) and ~25 kDa (Ab LC) size were cut from the gel and transferred to a sterile microcentrifuge tube. The proteins were extracted from the gel, digested with trypsin, and the gp41 and Tr-Env-CT samples were deglycosylated with Peptide-N-Glycosidase F. The tryptic peptides were analyzed by a Q Exactive^{TM} Hybrid Quadruple-Orbitrap^{TM} mass-spectrometer coupled to a EASY nLC1000 (ThermoFisher Scientific) liquid chromatography. The m/z ratio of the detected peptides was compared to a database containing the human proteome and HIV-1 isolate BH10 protein sequences, for gp41 and Tr-Env-CT, and the *Mus musculus* proteome for Ab LC.

Table S1. Proviral constructs expressing different variants of gp41.

	Construct	Gp41 variant	Description
Proviral	pCHIV	WT	Non-infectious plasmid expressing all HIV-1_{NL4-3} proteins except Nef, but cannot replicate because of the lack of the viral long-terminal repeat sequences[6].
	pCHIV ΔCT_{811}	ΔCT_{811}	pCHIV derivative expressing a gp41 protein variant with a 43 amino-acid truncation on its CT (L812*).
	pCHIV ΔCT_{750}	ΔCT_{750}	pCHIV derivative expressing a gp41 protein variant with a 104 amino-acid truncation on its CT (L751*).
	pCHIV $CRAC_{mut}$	L677I	pCHIV derivative expressing a gp41 protein variant with a disruption of the CRAC motif by a L677I substitution.
	pCHIV C762S	C76S	pCHIV derivating expressing a gp41 protein variant with a C762S substitution.
Env only	pCAGGS NL4-3 Env	WT	Plasmid expressing the HIV-1_{NL4-3} Env protein.

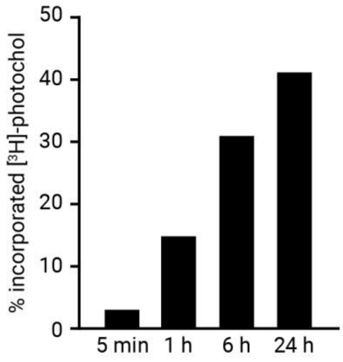

Figure S1. Percentage of [^{3}H]-photochol incorporated into the cells after different incubation times.

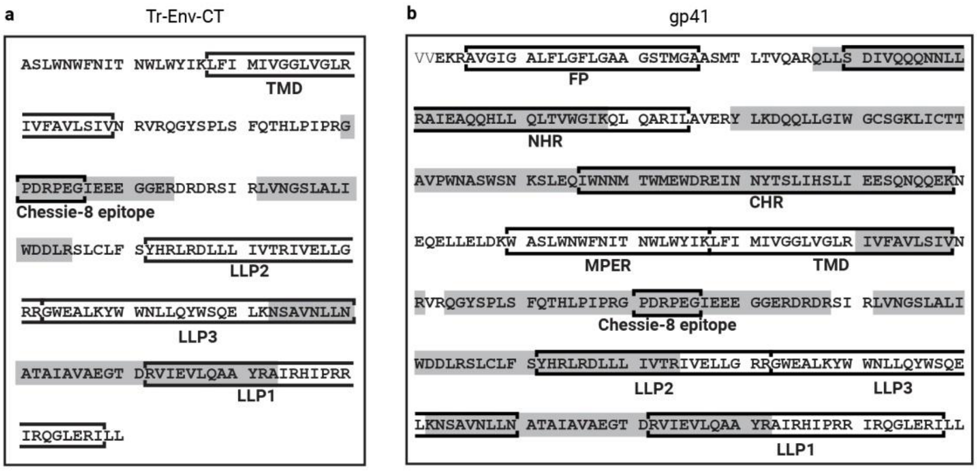

Figure S2. Proteomic analysis of proteins detected in anti-gp41 Western blot. (a) Peptides derived from the TR-Env-CT ~20 kDa protein band were detected by proteomic analysis. Different domains are marked in brackets. Shaded sequences represent the detected tryptic peptides. (b) Peptides derived from the gp41 ~41 kDa protein band were detected by proteomic analysis. Different domains are marked in brackets. Shaded sequences represent the detected tryptic peptides.

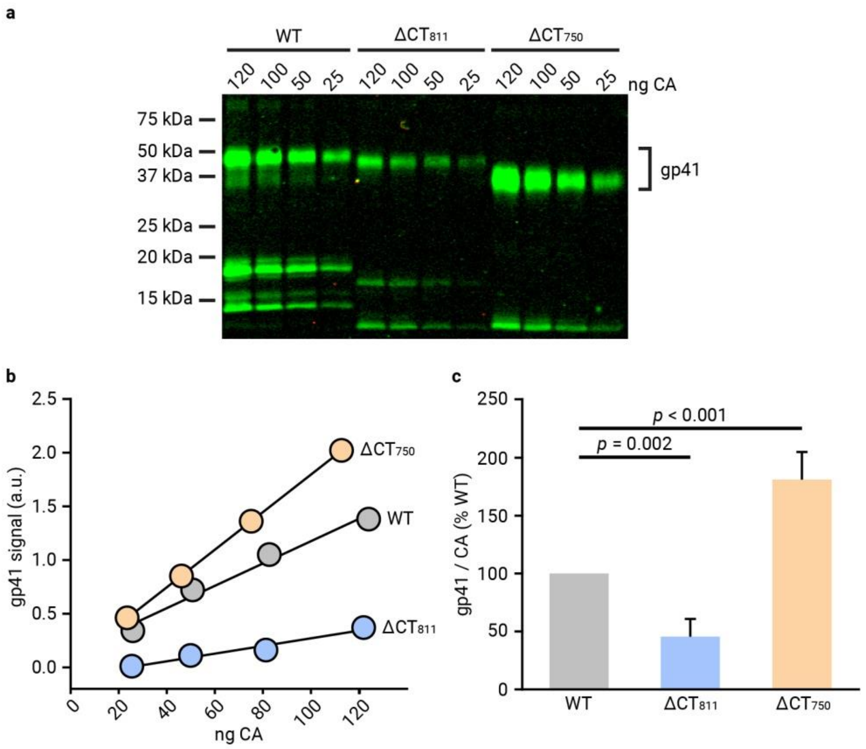

Figure S3. Effect of CT truncation in gp41 incorporation into the virion. (a) Representative Western blot of gp41 quantification. Volumes of purified viral particles corresponding to 120, 100, 50 and 25 ng of CA were loaded into an SDS-PAGE for each variant. The Western blot was developed against gp41. (b) Representative graph of gp41 signal blotted against the loaded ng of CA. The relative slopes of the linear curve fits of each variant were calculated. (c) The mean slopes of the variants were calculated relative to the wild type samples. The bars and whiskers represent the mean ± SD of experiments from three independent viral purifications (n = 3). Statistical significance was assessed by analysis of variance and Tukey test.

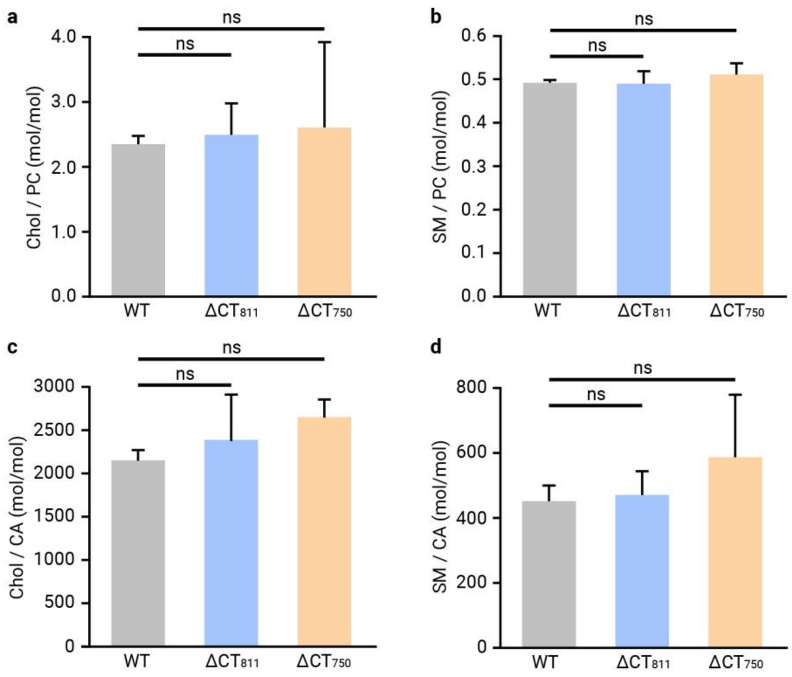

Figure S4. Effect of CT truncation in cholesterol and sphingomyelin (SM) recruitment into the virion. Cholesterol and SM content was analyzed by lipidomic analysis of viral particles containing wild-type or truncated gp41 variants. (a, b) Cholesterol and SM content relative to phosphatidylcholine (PC). The bars and whiskers represent the mean ± SD of experiments from three independent viral purifications. Statistical significance was assessed by analysis of variance and Tukey test. (c, d) Cholesterol and SM content relative to viral CA. The bars and whiskers represent the mean ± SD of experiments from three independent viral purifications (n = 3) with three replicas each. Statistical significance was assessed by analysis of variance and Tukey test.

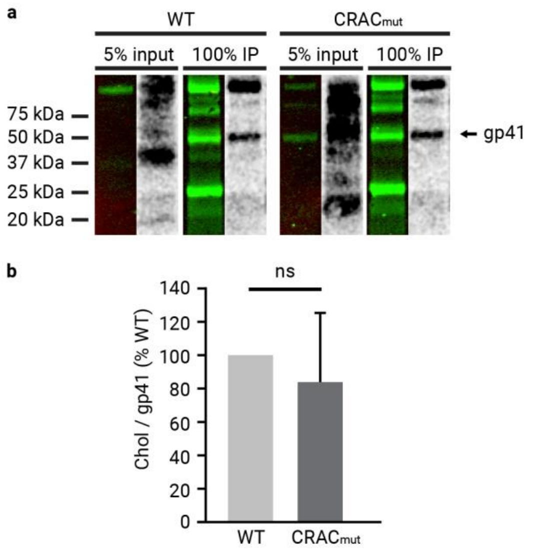

Figure S5. Effect of CRAC disruption (L677I) in gp41 interaction with chol in purified mature viral particles. (a) gp41 from input and immunoprecipitation (IP) from viral particles was detected by Western blot with the anti-gp41 chessie-8 antibody (green lane), and the interacting radioactively labeled lipid by autoradiography of the same membrane (gray lane). (b) Quantification of the cholesterol/protein signal ratio of the mutated $CRAC_{mut}$ gp41 protein compared to the wild-type gp41. The bars and whiskers represent the mean ± SD of experiments from three independent viral purifications (n = 3). Statistical significance was assessed by analysis of variance and Tukey test.

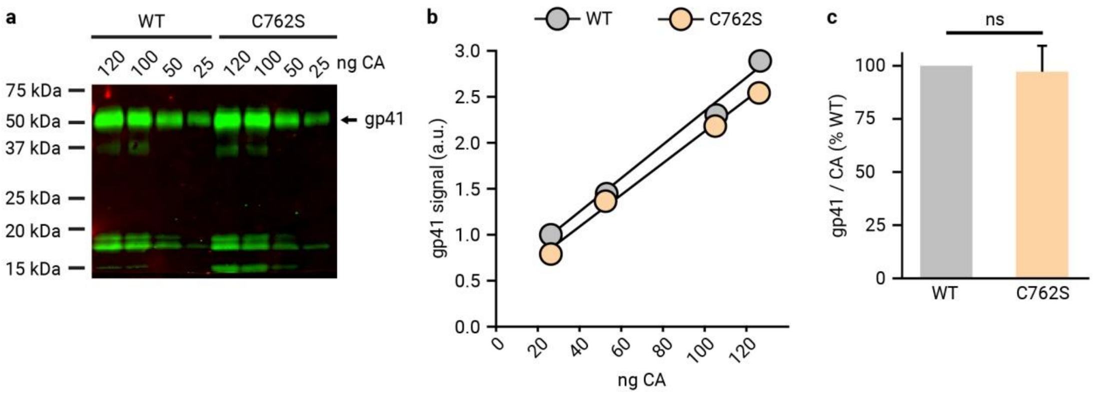

Figure S6. Effect of C762S mutation in gp41 incorporation into the virion. (a) Representative Western blot of gp41 quantification. Volumes of purified viral particles corresponding to 120, 100, 50 and 25 ng of CA were loaded into an SDS-PAGE for each variant. The Western blot was developed against gp41. (b) Representative graph of gp41 signal blotted against the loaded ng of CA. The relative slopes of the linear curve fits of each variant were calculated. (c) The mean slopes of the variants were calculated relative to the wild type samples. The bars and whiskers represent the mean ± SD of experiments from three independent viral purifications (n = 3). Statistical significance was assessed by t-test.

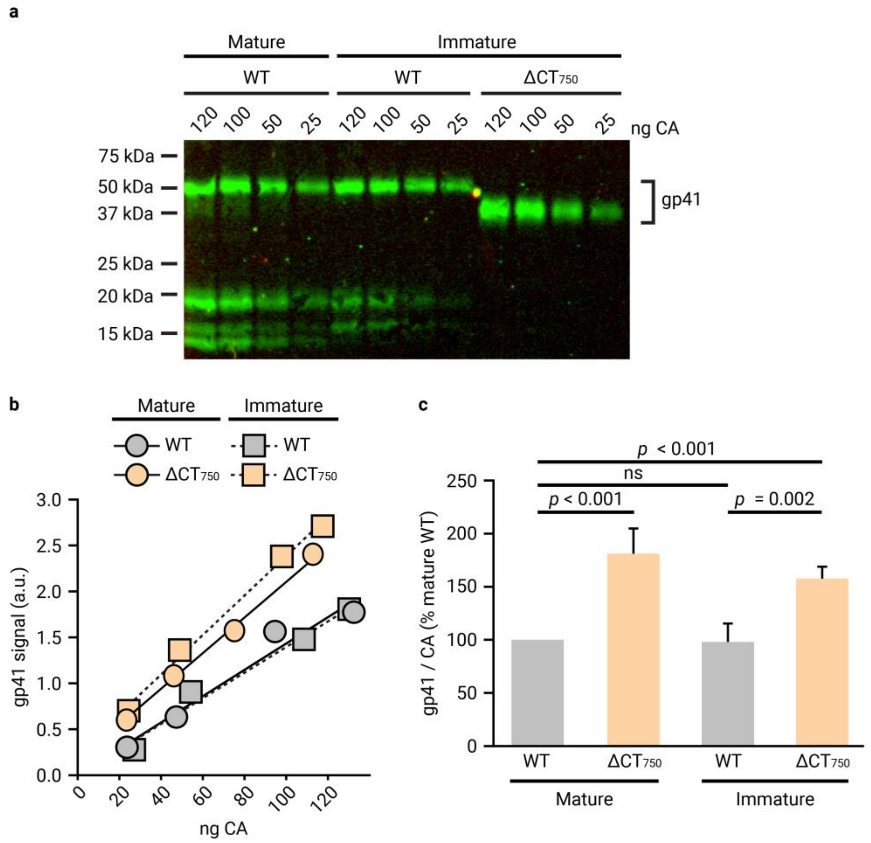

Figure S7. Effect of virus maturation and CT truncation in gp41 incorporation into the virion. (a) Representative Western blot of gp41 quantification. Volumes of purified viral particles corresponding to 120, 100, 50 and 25 ng of CA for mature viral particles, or Gag equivalent for immature viral particles were loaded into an SDS-PAGE for each variant. The Western blot was developed against gp41. (b) Representative graph of gp41 signal blotted against the loaded ng of CA or Gag equivalent. The relative slopes of the linear curve fits of each variant were calculated. (c) The mean slopes of the variants were calculated relative to the wild type samples. The bars and whiskers represent the mean ± SD of experiments from three independent viral purifications (n = 3). Statistical significance was assessed by analysis of variance and Tukey test.

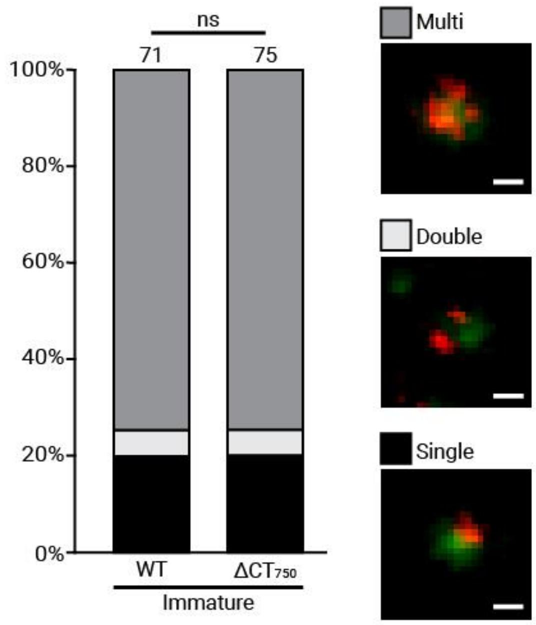

Figure S8. Env distribution patterns of immature virus particles with full-length and the truncated ΔCT_{750} gp41. The statistical significance was assessed by χ^2 test for independence at two degrees of freedom. The number of viral particles quantified in each preparation is represented above the bars (n > 70). Scale bars: 100 nm.

Supporting References

[1] C. Thiele, M. J. Hannah, F. Fahrenholz, W. B. Huttner, *Nat. Cell Biol.* **2000**, *2*, 42.
[2] M. Dettenhofer, X. F. Yu, *J. Virol.* **1999**, *73*, 1460.
[3] J. A. G. Briggs, M. N. Simon, I. Gross, H.-G. Kräusslich, S. D. Fuller, V. M. Vogt, M. C. Johnson, *Nat. Struct. Mol. Biol.* **2004**, *11*, 672.
[4] L. Carlson, J. A. G. Briggs, B. Glass, J. D. Riches, M. N. Simon, M. C. Johnson, B. Müller, K. Grünewald, H.-G. Kräusslich, *Cell Host Microbe* **2008**, *4*, 592.
[5] B. Brugger, B. B. Glass, P. Haberkant, I. Leibrecht, F. T. Wieland, H.-G. Krausslich, *Proc. Natl. Acad. Sci.* **2006**, *103*, 2641.
[6] B. Muller, J. Daecke, O. T. Fackler, M. T. Dittmar, H. Zentgraf, H.-G. Krausslich, B. Mu, J. Daecke, O. T. Fackler, M. T. Dittmar, H. Zentgraf, H. Kra, *J. Virol.* **2004**, *78*, 10803.
